# Decision-making in serial crystallography: a simple test to quickly determine whether sufficient data have been collected

**DOI:** 10.1107/S2059798326001324

**Published:** 2026-02-28

**Authors:** David von Stetten, Arwen R. Pearson

**Affiliations:** a European Molecular Biology Laboratory (EMBL) Notkestrasse 85 22760Hamburg Germany; b Institute for Nanostructure and Solid State Physics, Hamburg Centre for Ultrafast Imaging University of Hamburg HARBOR Building 610, Luruper Chaussee 149 22761Hamburg Germany; Global Phasing Ltd, United Kingdom

**Keywords:** serial crystallography, time-resolved crystallography, macromolecular crystallography, signal to noise

## Abstract

In standard rotational data collection for macromolecular crystallography, data are normally collected from a single crystal, and the resulting data processing delivers metrics for data completeness and signal to noise that are well established. However, in serial crystallography it can be difficult to assess quickly whether enough data have been recorded to deliver a well scaled and complete dataset with sufficient signal to noise to address the scientific question being asked. Completeness alone is not an appropriate metric, as a nominally complete dataset can be obtained with a much smaller number of images, and thus multiplicity, than is needed to produce a final dataset with well estimated merged intensity values. Insufficient data result in alarmingly reasonable processing statistics and plausible electron-density maps that contain almost no experimental signal, instead being dominated by the phases from the phasing model. We have therefore established a simple electron-density-based test to determine whether enough data have been collected, and implemented this in the autoprocessing pipeline at the T-REXX endstation on beamline P14 at PETRA III. Importantly, the results of this perturbed model real-space difference density (PMRDD) test help to guide decisions as to whether more data should be collected or whether the experimenter can move onto a new time-point or sample.

## Abstract 2

We describe a simple test to determine whether sufficient data have been collected during a serial crystallographic experiment, and its incorporation into the autoprocessing pipeline at the T-REXX endstation on beamline P14 at the PETRA III synchrotron.

## 1. Introduction

Serial crystallography is a diffraction method in which a large number of single microcrystals are delivered to the probe beam in random orientations (Chapman *et al.*, 2011 ▸). A number of sample-delivery options are available, depending on the source, beamline and experiment type (Martiel *et al.*, 2019 ▸). All serial experiments produce a series of diffraction patterns that are independent of each other in terms of orientation. During processing blank images are discarded and images containing diffraction spots, ‘hits’, are indexed and integrated. On the basis of the indexing, and the associated orientation matrix that describes the alignment of each crystal with respect to the laboratory frame, the integrated reflections can be assembled or ‘ordered’ into a 3D dataset. As each diffraction pattern is from a different crystal and has been independently processed, the resulting dataset contains a spread of unit-cell dimensions and varying intensity values for each reflection, for example as a result of varied crystal size, partiality and/or varying incident beam intensity. Next, a scaling algorithm attempts to converge on a good estimate of the true intensity of each reflection before a final merging step. Scaling removes outliers, applies scaling factors on a per-image basis and, in more complex processing pathways, can attempt to model the partiality of reflections on each frame. Scaling of serial data fundamentally relies on the law of large numbers, making the assumption that if the population of measurements (*i.e.* individual diffraction patterns) is large enough, the final mean value of the merged intensity will converge on the true value.

The danger in calculating electron-density maps from datasets with insufficient sampling of each reflection is that the resulting maps are dominated by the model phases. This phase bias can result in seductively beautiful but meaningless maps, leading the unwary crystallographer to assume the data are much better than they in fact are.

A key question during a serial diffraction experiment is therefore: when have sufficient data been collected to obtain a good enough estimate of the intensity of each reflection to allow confident interpretation of the resulting electron-density maps? The relevant statistical metric here is the multiplicity. This describes, on average, how often each reflection has been observed. However, this aggregate metric can be misleading, for example in cases where there is a strong preferential orientation of the individual crystals and some reflections have been measured many times, but others much less frequently. Other metrics usually used to assess data quality are less useful, or in the case of completeness (which only reports the proportion of possibly observable reflections that have been recorded at least once), actively misleading. A number of rules of thumb circulate in the serial community which attempt to help users decide when enough is enough in terms of data, but these can be effectively summarized as ‘more is always better’ (Schulz *et al.*, 2022 ▸; Mehrabi *et al.*, 2021 ▸). This is not a particularly helpful guideline for decision-making on the fly during data collection. We have therefore developed a perturbed model real-space difference density (PMRDD) test that can be run as part of the autoprocessing pipeline during serial experiments. This provides rapid feedback to users as to whether they have reached sufficient multiplicity, and thus sufficient signal to noise, to have overcome model phase bias. For time-resolved experiments, it can help to indicate whether the expected structural changes are likely to be visible in the electron-density map or whether additional data are needed.

## 2. Methods

### 2.1. Summary of the current T-REXX autoprocessing pipeline

T-REXX (‘time-resolved X-ray crystallography’) is an endstation on beamline P14 at the PETRA III synchrotron (DESY, Hamburg), operated by EMBL and dedicated to serial crystallographic experiments (von Stetten *et al.*, 2019 ▸). The autoprocessing pipeline implemented on T-REXX takes the form of a bash script generated by *MXCuBE* (Gabadinho *et al.*, 2010 ▸; Oscarsson *et al.*, 2019 ▸) on the basis of user-supplied information (starting model, cell and space-group parameters) and the type of serial experiment selected (static structure, burst mode, HARE data collection *etc.*; Schulz *et al.*, 2022 ▸). The script runs *CrystFEL* for peakfinding (*zaef*; White *et al.*, 2012 ▸), indexing (*XGandalf*; Gevorkov *et al.*, 2019 ▸), integration, scaling and merging (*partialator*; White *et al.*, 2016 ▸). *ambigator* is run automatically for those space groups where indexing ambiguities can occur (White *et al.*, 2016 ▸). Once scaling and merging are complete, the resulting merged intensities are written to MTZ format and passed to the *DIMPLE* pipeline (Wojdyr *et al.*, 2013 ▸), along with a user-provided model (PDB format). If there is a mismatch in space group or a large difference in unit-cell parameters from the user-provided model file, *DIMPLE* carries out molecular replacement using *Phaser* (McCoy *et al.*, 2007 ▸). *DIMPLE* finishes with four cycles of jelly-body and eight cycles of restrained refinement using *REFMAC*5 (Murshudov *et al.*, 2011 ▸) with twin refinement used in order to detect possible indexing ambiguities that are not successfully resolved by *ambigator*. *FFT* from the *CCP*4 suite (Agirre *et al.*, 2023 ▸) is then run to create 2*mF*_o_ − *DF*_c_ (FWT and PHWT) and *mF*_o_ − *DF*_c_ (DELFWT and PHDELWT) electron-density maps and these are passed to the ISPyB database (Dela­genière *et al.*, 2011 ▸), together with the MTZ and PDB file output by *REFMAC*5, additional data-quality plots and a ‘Table 1’-style summary of data-processing statistics. Users can then view these files and the resulting electron-density map and model through the EXI web interface (https://exi.embl-hamburg.de) using *UGLYMOL* (Wojdyr, 2017 ▸).

### 2.2. Model preparation for the perturbed model real-space difference density test (PMRDD)

To enable use of the PMRDD test in the T-REXX auto­processing pipeline, prior to loading into *MXCuBE* the user-supplied model is manually edited to remove a core, well ordered aromatic residue side chain that, if possible, is not close to the region of interest, for example the active site or ligand-binding site. Additionally, a second core aromatic residue is changed to a different rotamer that ideally does not clash with the rest of the model.

The user-supplied model is normally a refined model from a previous room-temperature (serial or single crystal) or cryo experiment on the same macromolecular target, or a sufficiently similar model from the wwPDB. Due to the nature of the experiments on T-REXX (*i.e.* time-resolved serial measurements), T-REXX users, so far, have never lacked a good starting model, but in principle this test is usable for serial experiments where the goal is *de novo* structure determination via molecular replacement.

### 2.3. Generation of test datasets to demonstrate utility of the PMRDD test

To demonstrate the impact of increasing multiplicity on electron-density map quality and to highlight the utility of the PMRDD test, and its robustness towards data collected in different kinds of serial experiments, we used several serial datasets collected using fixed targets at T-REXX at PETRA III (von Stetten *et al.*, 2019 ▸; Mehrabi *et al.*, 2020 ▸) and Cristallina at SwissFEL (Carrillo *et al.*, 2023 ▸), a microfluidic device at MASSIF-3 at ESRF (Von Stetten *et al.*, 2020 ▸; Monteiro *et al.*, 2020 ▸) and liquid jets at SPB/SFX at the European XFEL (Mancuso *et al.*, 2019 ▸; Schulz *et al.*, 2019 ▸) (Table 1 ▸). These were all processed using the standard T-REXX autoprocessing pipeline (identical processing parameters, aside from the XFEL datasets, which were introduced into the T-REXX pipeline post-integration) using *CrystFEL* v.0.10.2 with increasing numbers of images included in each merged dataset. For the CTXM dataset, test datasets were processed with and without the use of *ambigator* to resolve indexing ambiguities. Full processing statistics are provided as Supplementary Tables S1–S6.

## 3. Results

**Figure 1:**
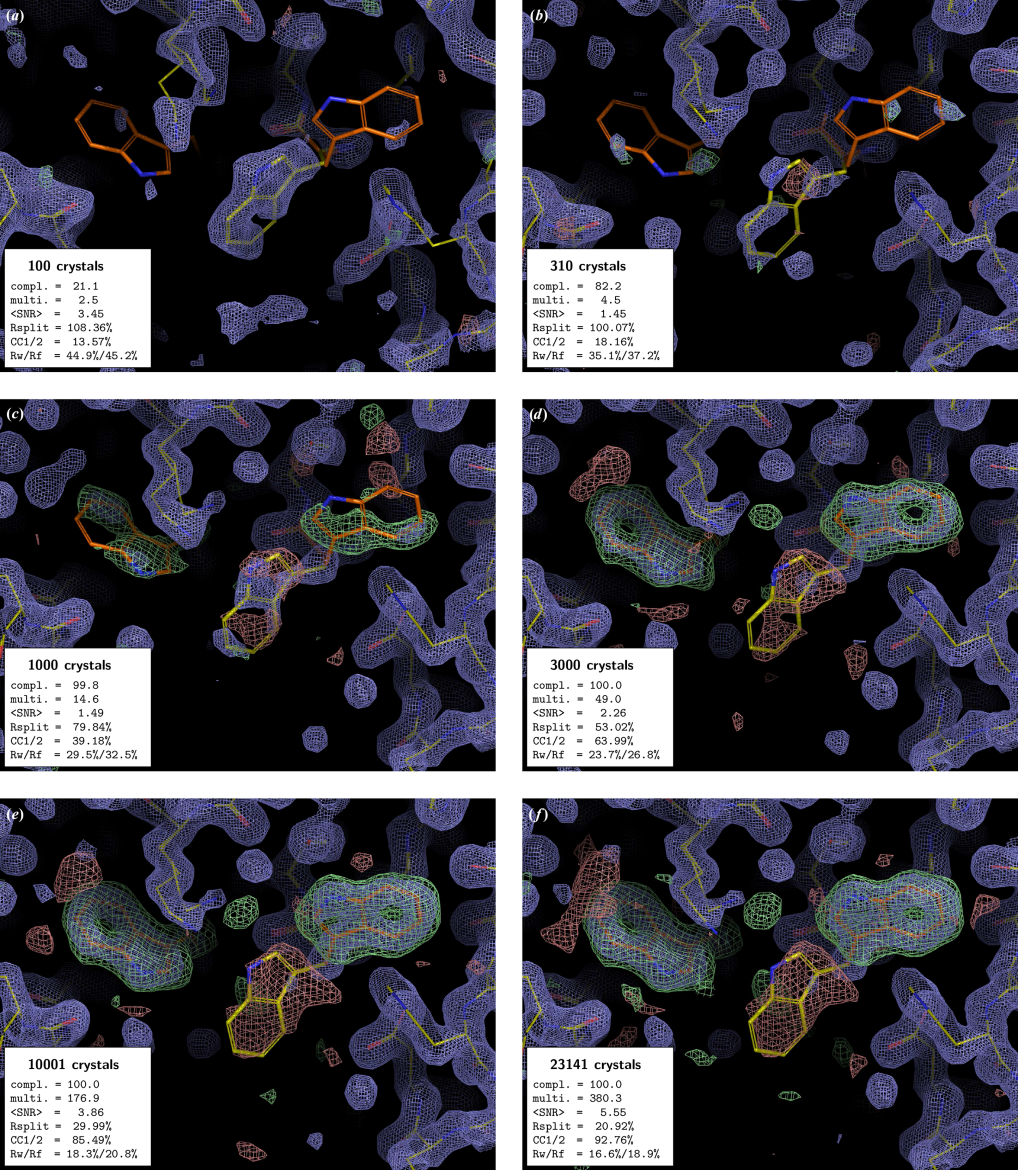
PMRDD test applied to a xylose isomerase serial dataset collected at T-REXX. Each panel shows a *PyMOL* (v.2.5.0; Schrödinger) generated equivalent of a *Coot* (Emsley *et al.*, 2010 ▸) screenshot, showing 2*mF*_o_ − *DF*_c_ electron-density maps contoured at 1.5 r.m.s.d. (blue mesh) and *mF*_o_ − *DF*_c_ electron-density maps contoured at ±3.0 r.m.s.d. (green, +3.0 r.m.s.d.; red, −3.0 r.m.s.d. mesh). In each panel, the ‘true’ starting model is shown as sticks with carbons coloured orange. The edited ‘PMRDD model’ is shown as sticks with carbons coloured yellow. In each panel, an inset box indicates the number of diffraction patterns included in the merge (number of crystals) and summarizes the key data-quality metrics [completeness (%), multiplicity, signal-to-noise ratio, *R*_split_ (%) (White *et al.*, 2012 ▸), CC_1/2_ (%) (Assmann *et al.*, 2016 ▸) and overall *R*_work_ and *R*_free_ (%)]. A complete table of data-quality statistics can be found in Supplementary Table S1.

In all examples, the PMRDD test shows clearly when sufficient data have been included to overcome model phase bias (Figs. 1 ▸–5 ▸ ▸ ▸ ▸ and Supplementary Figs. S1 and S2). This can be seen in the progressive appearance of positive difference electron density at the correct position for the moved and deleted residues, as well as negative difference density at the position of the moved residue.

For the beginning crystallographer, we emphasize the fact that even for datasets derived from a clearly insufficient number of diffraction patterns, the 2*mF*_o_ − *DF*_c_ electron-density maps appear to be surprisingly good. However, this is entirely due to model bias, where, due to the weakness of the experimental data, the 2*mF*_o_ − *DF*_c_ ‘filled’ map effectively becomes an *F*_c_ map (Murshudov *et al.*, 1997 ▸). This is clearly evident in the maps calculated with low numbers of diffraction patterns, where 2*mF*_o_ − *DF*_c_ electron density is present at the position of the residue that was moved to an incorrect position for the PMRDD test and absent for the residue that was deleted. Only once the number of diffraction patterns in a dataset is sufficient do the electron densities for these perturbed residues show up correctly in both the 2*mF*_o_ − *DF*_c_ and *mF*_o_ − *DF*_c_ maps.

Take, for example, the series of electron-density maps presented in Fig. 1 ▸. For Figs. 1 ▸(*a*) and 1 ▸(*b*) (100 and 310 crystals) little difference electron density is visible, and for the 100-crystal dataset reasonable 2*mF*_o_ − *DF*_c_ electron density (blue) is visible for the incorrect position of the central tryptophan, indicating that the electron-density map is dominated by the model phases. When 1000 crystals are included in the merge [Fig. 1 ▸(*c*)], the difference electron density (green/red) now clearly indicates a difference between the edited model and the data, with green positive difference electron density at the true positions of the moved/deleted tryptophans and red negative difference electron density at the incorrectly positioned tryptophan in the edited model. Is this sufficient? Certainly not yet, as the difference electron density is not recognisable as tryptophan, as it does not fully cover all the tryptophan atoms, indicating that model bias is still present. Next we can consider the 3000-crystal dataset [Fig. 1 ▸(*d*)]. Here, the positive difference electron density is clearly tryptophan-shaped for both tryptophans and there is little remaining 2*mF*_o_ − *DF*_c_ electron density at the incorrect tryptophan position in the edited model. However, the 2*mF*_o_ − *DF*_c_ density for the true positions of each tryptophan is still not fully contiguous for either tryptophan, and the associated global quality metrics shown in the inset are still relatively poor. By 10 001 crystals [Fig. 1 ▸(*e*)], the electron-density maps are now at a point that we would judge the data quality as sufficient to allow the confident modelling of a high-occupancy species into the map. However, it is clear from the global processing statistics that the addition of a further ∼13 000 crystals into the dataset [Fig. 1 ▸(*f*)] produces better final *R* factors, as well as merging statistics, although the electron-density maps no longer show a qualitative improvement when contoured at the same level.^1^ Whether the data are now good enough to support confident modelling of a low-occupancy species depends on the specific experiment.

The PMRDD test works equally well, regardless of resolution and space group. In all cases it can be seen that the data are technically complete long before there is clear difference density overcoming the model bias.

**Figure 2:**
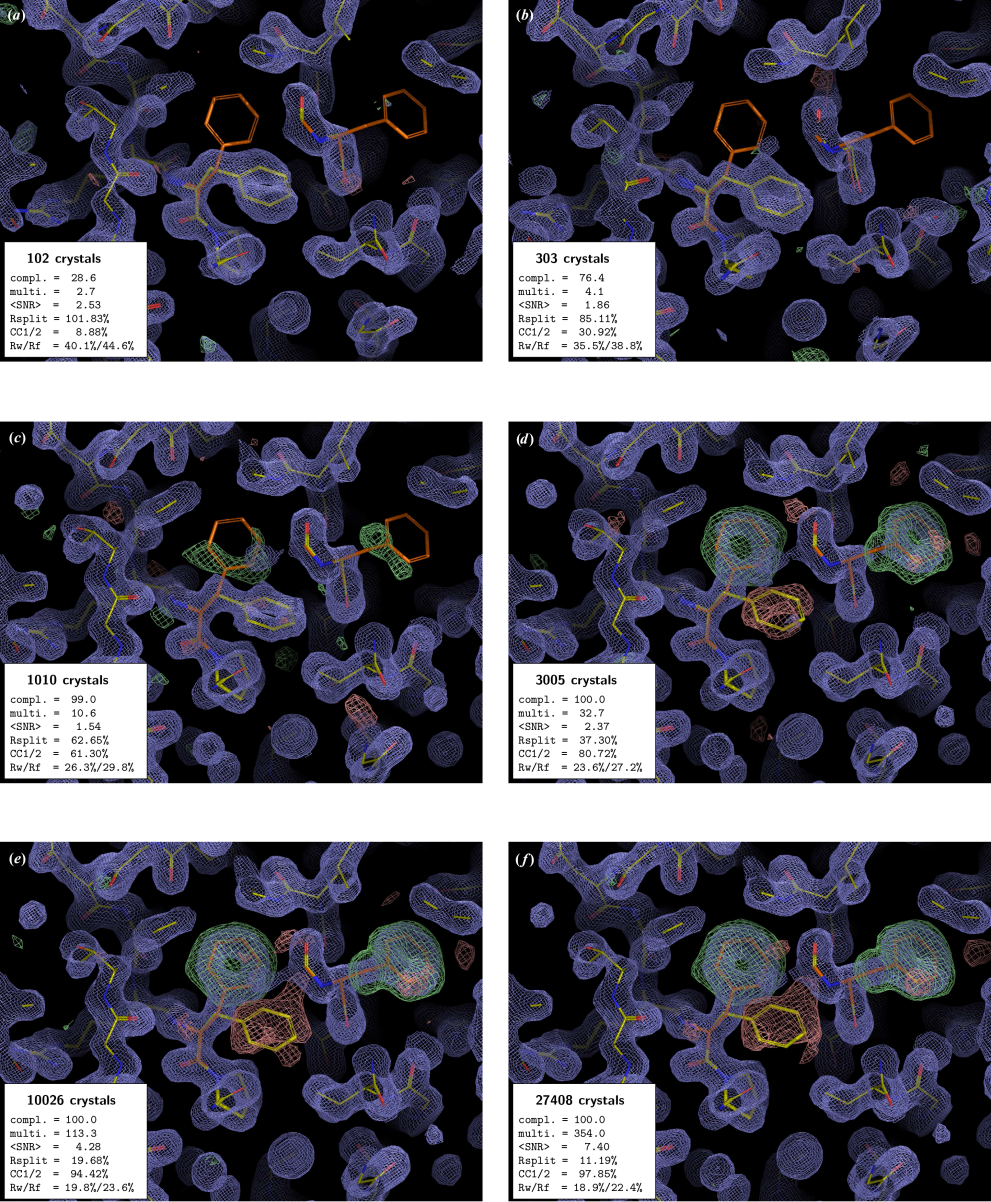
PMRDD test applied to a CTXM β-lactamase serial dataset collected at T-REXX, with the use of *ambigator* during the data processing. The panels, electron-density maps and colouring are all as in Fig. 1 ▸. A complete table of data-quality statistics can be found in Supplementary Table S2.

A few interesting additional observations can be made from these test data. Firstly, the use of *ambigator* to resolve indexing ambiguities has a noticeable positive effect on the quality of the difference density, even though for the CTXM datasets with up to 1000 diffraction patterns *ambigator* fails to resolve the indexing ambiguity, and consequently *REFMAC*5 determines these datasets as ‘twinned’ (compare Fig. 2 ▸ and Supplementary Fig. S1 and Supplementary Tables S2 and S6). However, for some datasets with a low number of crystals (*e.g.* 100- and 300-crystal datasets of xylose isomerase), *REFMAC*5 reports multiple twin domains (despite the absence of an indexing ambiguity) due to the rather poor data. Secondly, the data recorded using the 3DMixD microfluidic chip show clear difference density for the correct structure at a much lower number of crystals than the data recorded using fixed targets at synchrotrons or GDVN nozzles at the European XFEL. This could be due to the possibility for the crystals to rotate during X-ray exposure (∼5 ms residence time in the beam) as they flow through the microfluidic device. This would result in the measurement of more fully recorded reflections, potentially leading to more stable scaling and convergence to a good estimate of their intensities at lower multiplicity. However, it is also possible that the high symmetry of ADC is contributing. Further work will be needed to distinguish these possibilities.

## 4. Summary

The PMRDD test is a robust and simple test to help users decide when sufficient data have been collected in a serial experiment. It is easy to implement, either as a standalone test by an individual user (by simply deleting/moving a well ordered residue that is not expected to change position during the experiment prior to phasing and map calculation) or within a data-processing pipeline at a synchrotron or XFEL facility by providing a PDB file that has been modified as described here. It is useful at all resolutions tested and, maybe most importantly, is conceptually easy for novice users to grasp.

## Supplementary Material

Supplementary Figures and Tables. DOI: 10.1107/S2059798326001324/von5005sup1.pdf

## Figures and Tables

**Figure 3:**
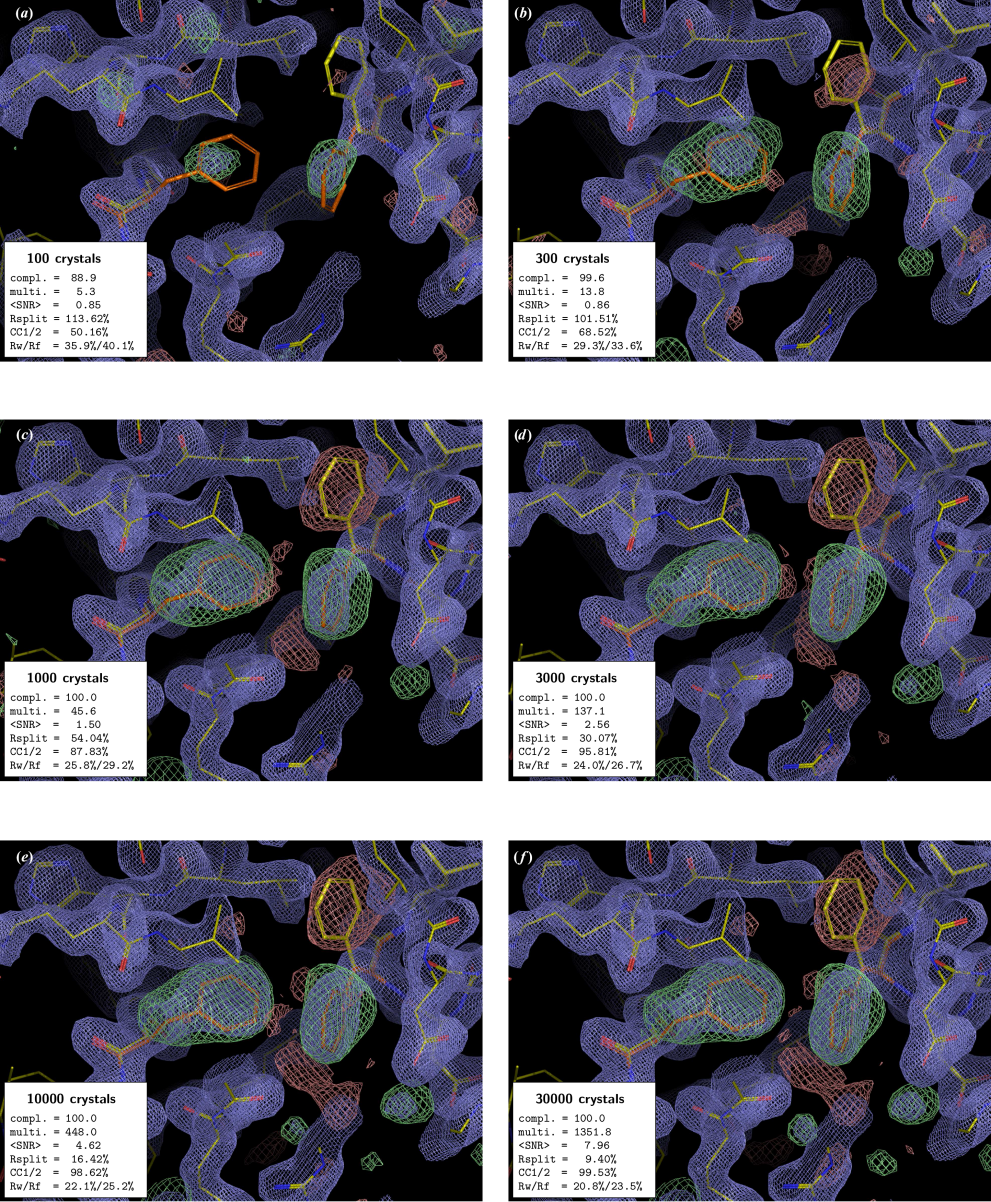
PMRDD test applied to an aspartate decarboxylase (ADC) serial dataset collected at MASSIF-3, ESRF. The panels, electron-density maps and colouring are all as in Fig. 1 ▸. A complete table of data-quality statistics can be found in Supplementary Table S3.

**Figure 4:**
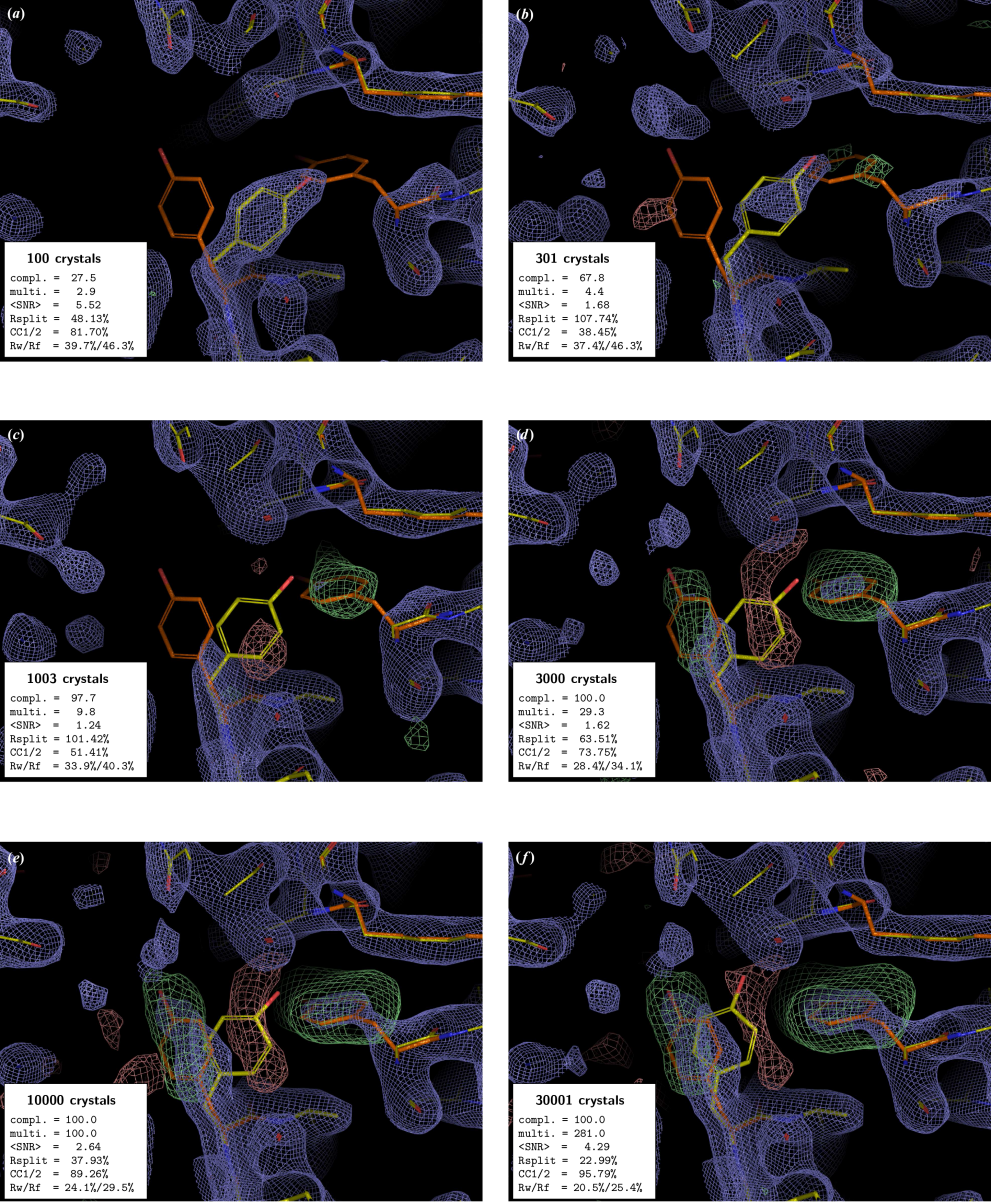
PMRDD test applied to a phytochrome A (phyA) serial dataset collected at the European XFEL. The panels, electron-density maps and colouring are all as in Fig. 1 ▸. A complete table of data-quality statistics can be found in Supplementary Table S4.

**Figure 5:**
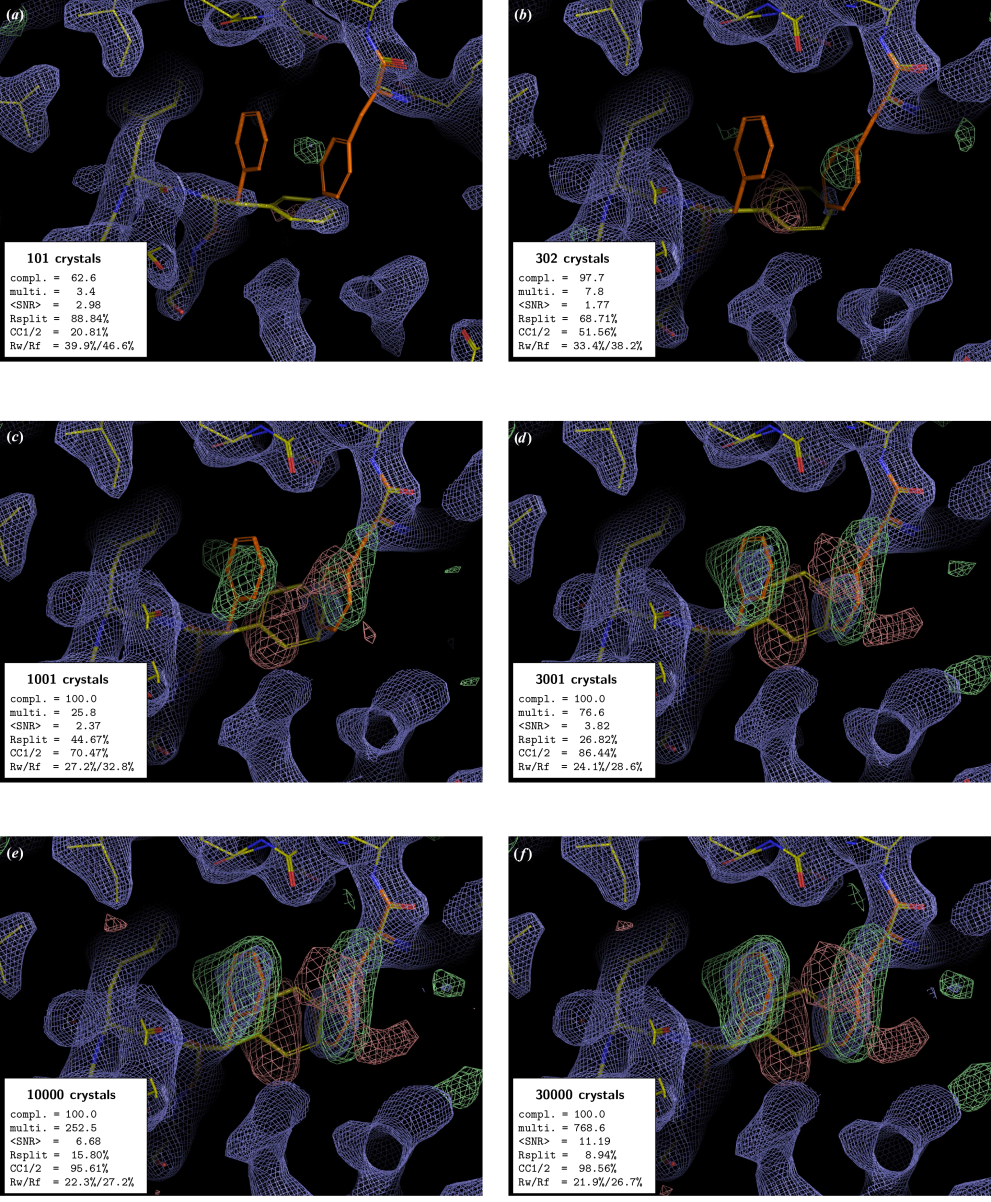
PMRDD test applied to a photoactivated adenylate kinase (OaPAC) dataset collected at Cristallina, SwissFEL. The panels, electron-density maps and colouring are all as in Fig. 1 ▸. A complete table of data-quality statistics can be found in Supplementary Table S5.

**Table 1:** Summary of test datasets used to demonstrate the PMRDD test

Protein	Endstation, source	Sample-delivery method	Resolution (Å)	Space group	No. of crystals per test dataset†
Xylose isomerase‡	T-REXX, PETRA III	HARE chip (fixed target)	1.7	*I*222	100, 310, 1000, 3000, 10001, 23141
CTXM‡ (with *ambigator*)	T-REXX, PETRA III	HARE chip (fixed target)	1.7	*P*3_2_21	102, 303, 1010, 3005, 10026, 27408
CTXM‡ (without *ambigator*)	T-REXX, PETRA III	HARE chip (fixed target)	1.7	*P*3_2_21	101, 297, 1008, 3000, 10009, 27421
ADC§	MASSIF-3, ESRF	3DMixD (microfluidic chip)	1.8	*P*6_1_22	100, 300, 1000, 3000, 10000, 30000
PhyA¶	SPB/SFX, EuXFEL	GDVN liquid jet	2.2	*P*2_1_	101, 301, 1003, 3000, 10000, 30001
OaPAC††	Cristallina, SwissFEL	MISP chip (fixed target)	2.2	*P*2_1_2_1_2	101, 302, 1001, 3001, 10000, 30000

† The number of images per test dataset is not completely uniform due to the way in which the *partialator* programme within *CrystFEL* rejects images during the scale and merge step.

‡ The xylose isomerase data and CTXM β-lactamase data have previously been reported in Schulz *et al.* (2025 ▸).

§ The aspartate decarboxylase data have previously been reported in Monteiro *et al.* (2020 ▸).

¶ The phytochrome A data have previously been reported in Nagano *et al.* (2025 ▸).

†† The photoactivated adenylate cyclase data are unpublished and are used here with the kind permission of Sofia Kapetanaki.

## Data Availability

The xylose isomerase data and CTXM β-lactamase data have been previously reported in Schulz *et al.* (2025): PDB entries 9g5n and 9g7w, respectively. The aspartate decarboxylase data have been previously reported in Monteiro *et al.* (2020), PDB entry 6rxh, and the raw data are available at https://www.cxidb.org/id-147.html, dataset chip3_ADC5_12_3mm.tgz. The phytochrome A data have been previously reported in Nagano *et al.* (2025). The OaPAC data are unpublished and are used here by kind permission of Sofia Kapetanaki.

## Footnotes

1 We note that comparison of electron-density maps should be done with care, as the variance of these maps will of course change as more crystals are added into the dataset. This means that comparing maps nominally contoured at the same r.m.s.d. is not a truly valid comparison.
