# Supplementary material for: Decision-making in serial crystallography: a simple test to quickly determine whether sufficient data have been collected
Source: Acta Crystallogr D Struct Biol. 2026 Feb 28;82(Pt 3):227–36. doi: 10.1107/S2059798326001324 (PMC12954858; doi:10.1107/S2059798326001324)
Supplement: Supplementary file 1 [file d-82-00227-sup1.pdf]

STRUCTURAL BIOLOGY

Volume 82 (2026)

Supporting information for article:

Decision-making in serial crystallography: a simple test to quickly determine whether sufficient data have been collected

David von Stetten and Arwen R. Pearson

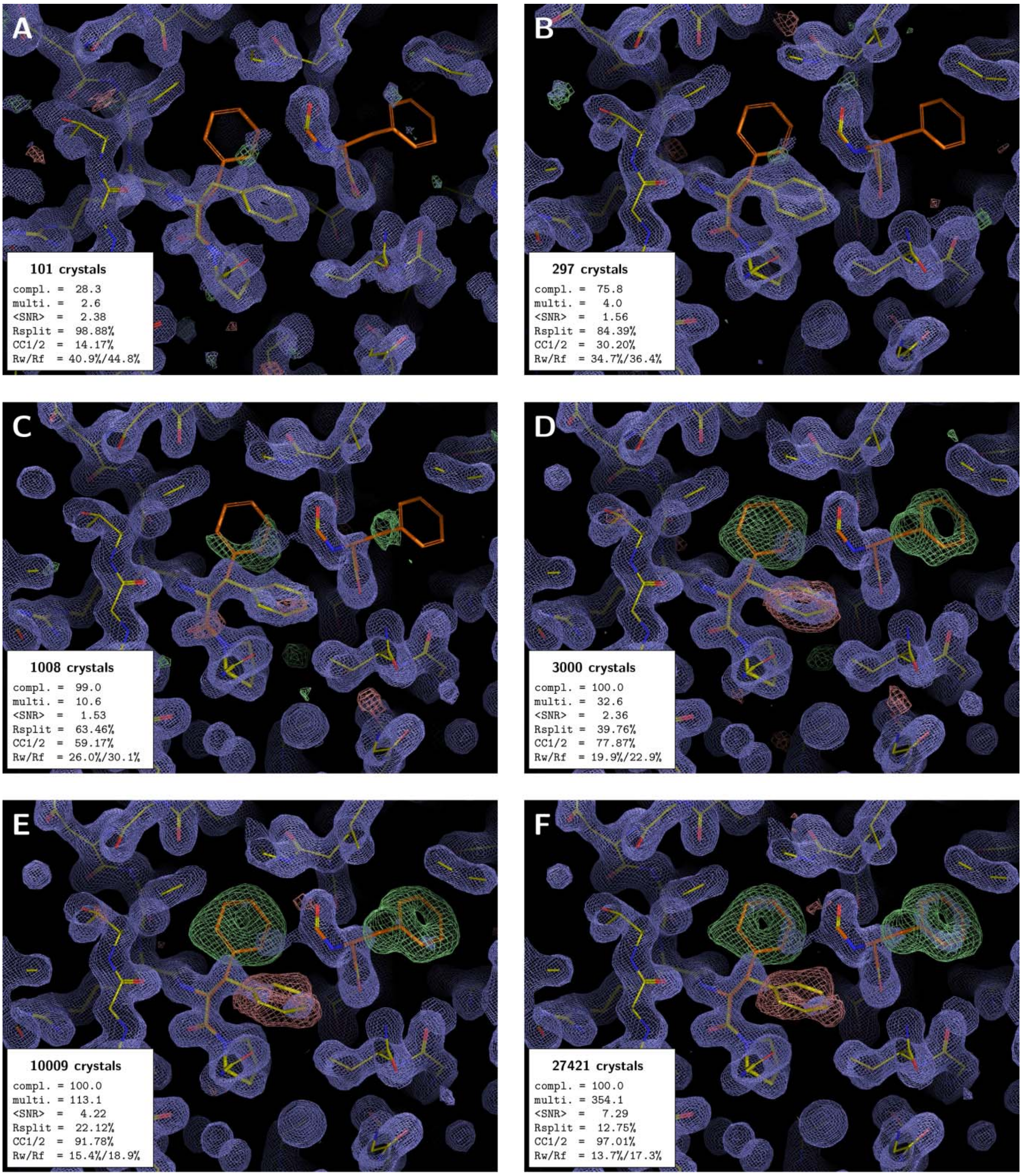

Figure S1 PMRDD test applied to a CTXM beta lactamase serial dataset collected at T-REXX, without the use of *ambigator* during data processing. Each panel shows a Pymol (v2.5.0) (Schrödinger, LLC, 2015) generated equivalent of a COOT (Emsley *et al.*, 2010) screenshot, showing $2mF_o$-DF_c electron density maps contoured at 1.5 r.m.s.d. (blue mesh) and mF_o-DF_c electron density maps contoured at ± 3.0 r.m.s.d. (green + 3.0 r.m.s.d. and red -3.0 r.m.s.d. mesh). In each panel, the “true” starting model is shown as sticks with carbons coloured orange. The edited “PMRDD model” is shown as sticks with carbons coloured yellow. In each panel, an inset box indicates the number of diffraction patterns included in the merge (number of crystals) and summarises the key data quality metrics (completeness [%], multiplicity, signal-to-noise ratio, R_{split} [%](White *et al.*, 2012), CC1/2

[%] (Assmann *et al.*, 2016), and overall R_{work} and R_{free} [%]). A complete table of data quality statistics can be found in the supplementary data Table S6.

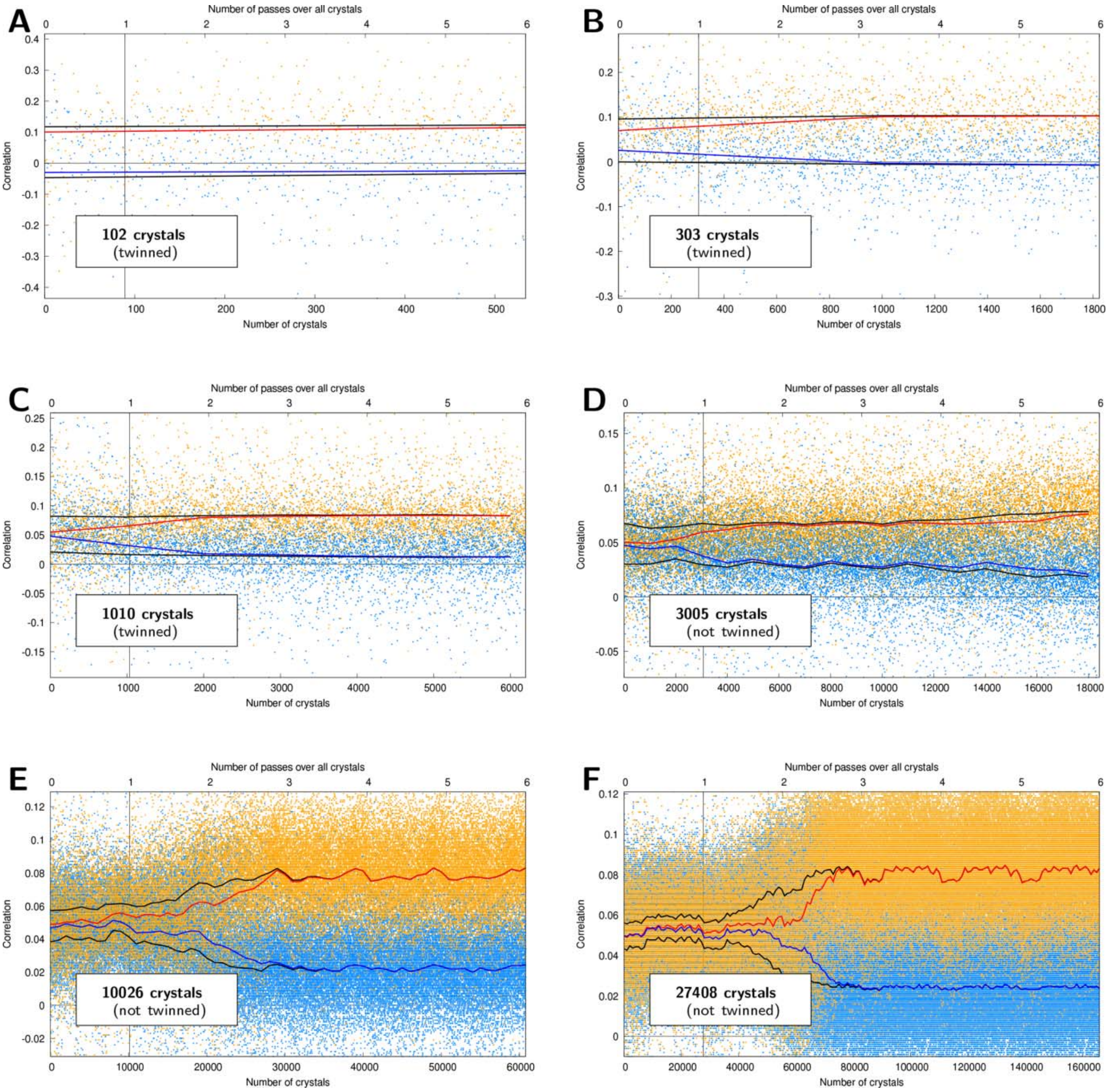

Figure S2 *Ambigator* correlation plots for the CTXM beta lactamase serial dataset collected at T-REXX, corresponding to the electron density images shown in Figure 2. Each panel contains an inset indicating the number of diffraction patterns included in the merge (number of crystals) and indicates whether REFMAC identifies the data as twinned or not. At low numbers of crystals included in the merged dataset, the indexing ambiguity cannot be resolved by *ambigator* and REFMAC identifies the data as twinned. By ~3000 crystals in the merge (Panel D) *ambigator* sufficiently resolves the indexing ambiguity such that REFMAC no longer identifies the data as twinned.

Table S1 Data processing statistics for the xylose isomerase dataset (Schulz *et al.*, 2025). Data were collected at the T-REXX endstation on EMBL beamline P14 at PETRA III. Data were collected at 295 K. Samples were delivered to the X-ray beam using HARE-Chips (Mehrabi *et al.*, 2020).

**Data collection**						
# diffraction patterns	100	310	1000	3000	10001	23141
Space group	I222					
Unit cell dimensions (Å, °)	94.2, 103.0, 99.2; 90.0, 90.0, 90.0					
Resolution range (Å)	71.4 – 1.70	71.4 – 1.70	71.4 – 1.70	71.4 – 1.70	71.4 – 1.70	71.4 – 1.70
	(1.76 – 1.70)	(1.76 – 1.70)	(1.76 – 1.70)	(1.76 – 1.70)	(1.76 – 1.70)	(1.76 – 1.70)
Wilson B factor (Å^2)	20.0	21.4	20.4	20.2	20.6	20.2
# measured reflections	28305	197921	777629	2616262	9440232	20298653
# unique reflections	11285	43880	53241	53373	53373	53373
Completeness (%)	21.1 (9.7)	82.2 (65.0)	99.8 (99.2)	100.0 (100.0)	100.0 (100.0)	100.0 (100.0)
Multiplicity	2.5 (2.2)	4.5 (3.1)	14.6 (8.6)	49.0 (28.9)	176.9 (104.2)	380.3 (224.3)
Signal-noise-ratio	3.5 (1.1)	1.4 (0.6)	1.5 (0.6)	2.3 (1.2)	3.9 (2.2)	5.5 (3.3)
Rsplit (%)	108.4 (164.8)	100.1 (207.8)	79.8 (176.4)	53.0 (86.8)	30.0 (48.6)	20.9 (34.0)
CC* (%)	48.9 (NaN)	55.4 (34.0)	75.0 (45.9)	88.3 (70.8)	96.0 (88.2)	98.1 (93.0)
CC1/2 (%)	13.6 (45.8)	18.2 (6.1)	39.2 (11.8)	64.0 (33.4)	85.5 (63.6)	92.8 (76.2)
**Refinement**						
Resolution range (Å)	71.5 – 1.56	71.5 – 1.56	71.5 – 1.70	71.5 – 1.70	71.5 – 1.70	71.5 – 1.70

Mean B (Å^2)	10.7	12.5	14.5	15.1	15.5	15.2
R_{work} (%)	44.9	35.1	29.5	23.7	18.3	16.6
R_{free} (%)	45.2	37.2	32.5	26.8	20.8	18.9
# Twin domains[2]	6	4	0	0	0	0

[1] Numbers in parentheses refer to the highest resolution shell

[2] As discussed in §3 of the main text, twin domains are erroneously detected by REFMAC due to the poor data quality.

Table S2 Data processing statistics for the CTXM beta-lactamase dataset using ambigator (Schulz *et al.*, 2025). Data were collected at the T-REXX endstation on EMBL beamline P14 at PETRA III. Data were collected at 295 K. Samples were delivered to the X-ray beam using HARE-Chips (Mehrabi *et al.*, 2020).

**Data collection**						
# diffraction patterns	102	303	1010	3005	10026	27408
Space group	$P3_221$					
Unit cell dimensions (Å, °)	41.9, 41.9, 232.8; 90.0, 90.0, 120.0					
Resolution range (Å)	116.3 – 1.70 (1.76 – 1.70)	116.3 – 1.70 (1.76 – 1.70)	116.3 – 1.70 (1.76 – 1.70)	116.3 – 1.70 (1.76 – 1.70)	116.3 – 1.70 (1.76 – 1.70)	116.3 – 1.70 (1.76 – 1.70)
Wilson B factor (Å^2)	24.4	24.6	24.6	24.6	24.3	24.1
# measured reflections	20776	85778	289412	897047	3111476	9718004
# unique reflections	7838	20979	27178	27452	27454	27455
Completeness (%)	28.6 (15.2)	76.4 (58.7)	99.0 (97.5)	100.0 (100.0)	100.0 (100.0)	100.0 (100.0)
Multiplicity	2.7 (2.2)	4.1 (3.0)	10.6 (6.5)	32.7 (19.7)	113.3 (68.7)	354.0 (214.2)
Signal-noise-ratio	2.5 (0.1)	1.9 (0.7)	1.5 (0.4)	2.4 (0.6)	4.3 (1.1)	7.4 (2.2)
Rsplit (%)	101.8 (-266.0)	85.1 (308.8)	62.6 (313.5)	37.3 (174.4)	19.7 (90.7)	11.2 (45.2)
CC* (%)	40.4 (54.0)	68.7 (NaN)	87.2 (32.4)	94.5 (50.3)	98.6 (81.1)	99.5 (94.8)
CC1/2 (%)	8.9 (17.0)	30.9 (0.6)	61.3 (5.5)	80.7 (14.5)	94.4 (48.9)	97.8 (81.5)
**Refinement**						

Resolution range (Å)	77.6 – 1.70	77.6 – 1.70	77.6 – 1.70	77.6 – 1.70	77.6 – 1.70	77.6 – 1.70
Mean B ($Å^2$)	18.5	20.8	21.2	22.8	22.9	22.8
R_{work} (%)	40.1	35.5	26.3	23.6	19.8	18.9
R_{free} (%)	44.6	38.8	29.8	27.2	23.6	22.4
# Twin domains[2]	2	2	2	0	0	0

[1] Numbers in parentheses refer to the highest resolution shell

[2] As discussed in §3 of the main text, twin domains are erroneously detected by REFMAC when *ambigator* is unable to resolve the indexing ambiguity that arises during processing of a serial dataset.

Table S3 Data processing statistics for the aspartate decarboxylase (ADC) dataset (Monteiro *et al.*, 2020). Data were collected at the MASSIF-3 beamline at ESRF. Data were collected at 295 K. Samples were delivered to the X-ray beam using the 3DMiXD microfluidic chip (Monteiro *et al.*, 2020).

**Data collection**						
# diffraction patterns	100	300	1000	3000	10000	30000
Space group	$P6_122$					
Unit cell dimensions (Å, °)	72.0, 72.0, 216.8; 90.0, 90.0, 120.0					
Resolution range (Å)	108.7 – 1.80 (1.86 – 1.80)	108.7 – 1.80 (1.86 – 1.80)	108.7 – 1.80 (1.86 – 1.80)	108.7 – 1.80 (1.86 – 1.80)	108.7 – 1.80 (1.86 – 1.80)	108.7 – 1.80 (1.86 – 1.80)
Wilson B factor ($Å^2$)	26.8	27.5	27.4	27.2	27.3	26.9
# measured reflections	150022	440087	1457661	4384787	14327015	43227855
# unique reflections	28438	31860	31971	31979	31979	31979
Completeness (%)	88.9 (76.3)	99.6 (99.1)	100.0 (100.0)	100.0 (100.0)	100.0 (100.0)	100.0 (100.0)
Multiplicity	5.3 (3.6)	13.8 (8.5)	45.6 (27.7)	137.1 (83.8)	448.0 (274.1)	1351.8 (827.7)
Signal-noise-ratio	0.8 (0.1)	0.9 (0.1)	1.5 (0.1)	2.6 (0.3)	4.6 (0.6)	8.0 (1.0)
Rsplit (%)	113.6 (14991.0)	101.5 (1285.3)	54.0 (739.1)	30.1 (367.7)	16.4 (204.2)	9.4 (111.1)
CC* (%)	81.7 (44.1)	90.2 (17.7)	96.7 (20.6)	98.9 (33.2)	99.7 (52.7)	99.9 (76.4)
CC1/2 (%)	50.2 (10.8)	68.5 (1.6)	87.8 (2.2)	95.8 (5.8)	98.6 (16.1)	99.5 (41.1)
**Refinement**						
Resolution range (Å)	62.4 – 1.80	62.4 – 1.80	62.4 – 1.80	62.4 – 1.80	62.4 – 1.80	54.1 – 1.80

Mean B (Å^2)	21.4	30.6	33.5	31.4	30.3	29.6
R_{work} (%)	35.9	29.3	25.8	24.0	22.1	20.8
R_{free} (%)	40.1	33.6	29.2	26.7	25.2	23.5

[1] Numbers in parentheses refer to the highest resolution shell

Table S4 Data processing statistics for the phytochrome A (phyA) dataset (Nagano *et al.*, 2025). Data were collected at the SPB/SFX instrument at European XFEL. Data were collected at 295 K. Samples were delivered to the X-ray beam using a GDVN (Schulz *et al.*, 2019).

**Data collection**						
# diffraction patterns	100	301	1003	3000	10000	30001
Space group	$P2_1$					
Unit cell dimensions (Å, °)	56.5, 115.0, 69.8; 90.0, 92.7, 90.0					
Resolution range (Å)	16.9 – 2.20 (2.28 – 2.20)[1]	18.5 – 2.20 (2.28 – 2.20)	20.3 – 2.20 (2.28 – 2.20)	20.5 – 2.20 (2.28 – 2.20)	20.5 – 2.20 (2.28 – 2.20)	20.5 – 2.20 (2.28 – 2.20)
Wilson B factor ($Å^2$)	-18.2	16.1	44.8	39.8	40.7	41.3
# measured reflections	36315	133496	431158	1322720	4512693	12680322
# unique reflections	12421	30590	44102	45124	45132	45132
Completeness (%)	27.5 (21.7)	67.8 (59.0)	97.7 (96.5)	100 (100)	100 (100)	100 (100)
Multiplicity	2.9 (2.7)	4.4 (3.7)	9.8 (7.5)	29.3 (22.1)	100.0 (75.1)	281.0 (212.3)
Signal-noise-ratio	5.5 (1.6)	1.7 (0.0)	1.2 (0.4)	1.6 (0.3)	2.6 (0.5)	4.3 (0.8)
Rsplit (%)	48.1 (165.8)	107.7 (-437.0)	101.4 (772.9)	63.5 (414.9)	37.9 (200.2)	23.0 (125.2)
CC* (%)	94.8 (39.8)	74.5 (39.0)	82.4 (52.7)	92.1 (55.5)	97.1 (73.8)	98.9 (78.1)
CC1/2 (%)	81.7 (8.6)	38.5 (8.2)	51.4 (16.1)	73.8 (18.2)	89.3 (37.5)	95.8 (43.8)
**Refinement**						
Resolution range (Å)	20.5 – 2.11	19.9 – 2.14	20.3 – 2.20	20.5 – 2.20	20.5 – 2.20	20.5 – 2.20
Mean B ($Å^2$)	30.2	36.1	44.8	45.3	46.7	45.8

R_{work} (%)	39.7	37.4	33.9	28.4	24.1	20.5
R_{free} (%)	46.3	46.3	40.3	34.1	29.5	25.4
# Twin domains[2]	2	2	0	0	0	0

[1] Numbers in parentheses refer to the highest resolution shell

[2] As discussed in §3 of the main text, twin domains are erroneously detected by REFMAC due to the poor data quality.

Table S5 Data processing statistics for the photoactivated adenylate cyclase (OaPAC) dataset. Data were collected at the Cristallina endstation on the ARAMIS beamline at SwissFEL. Data were collected at 295 K. Samples were delivered to the X-ray beam using MISP-Chips (Carrillo *et al.*, 2023).

**Data collection**						
# diffraction patterns	101	302	1001	3001	10000	30000
Space group	$P2_12_12$					
Unit cell dimensions (Å, °)	103.0, 55.0, 73.0; 90.0, 90.0, 90.0					
Resolution range (Å)	42.0 – 2.20 (2.28 – 2.20)	43.9 – 2.20 (2.28 – 2.20)	43.9 – 2.20 (2.28 – 2.20)	43.9 – 2.20 (2.28 – 2.20)	43.9 – 2.20 (2.28 – 2.20)	43.9 – 2.20 (2.28 – 2.20)
Wilson B factor ($Å^2$)	40.3	37.2	35.4	34.8	34.7	35.3
# measured reflections	46161	165193	560286	1665912	5491412	16719231
# unique reflections	13616	21254	21750	21752	21752	21752
Completeness (%)	62.6 (48.4)	97.7 (95.8)	100.0 (100.0)	100.0 (100.0)	100.0 (100.0)	100.0 (100.0)
Multiplicity	3.4 (2.7)	7.8 (5.5)	25.8 (18.0)	76.6 (53.5)	252.5 (176.6)	768.6 (537.3)
Signal-noise-ratio	3.0 (1.2)	1.8 (0.8)	2.4 (1.1)	3.8 (1.9)	6.7 (3.4)	11.2 (5.8)
Rsplit (%)	88.8 (-6579.5)	68.7 (160.5)	44.7 (95.8)	26.8 (52.2)	15.8 (28.1)	8.9 (16.4)
CC* (%)	58.7 (48.1)	82.5 (59.4)	90.9 (74.5)	96.3 (90.7)	98.9 (96.9)	99.6 (98.9)
CC1/2 (%)	20.8 (13.1)	51.6 (21.4)	70.5 (38.5)	86.4 (69.8)	95.6 (88.4)	98.6 (95.7)
**Refinement**						
Resolution range (Å)	43.9 – 2.20	43.9 – 2.20	43.9 – 2.20	43.9 – 2.20	43.9 – 2.20	43.9 – 2.20

Mean B (Å^2)	38.7	43.0	45.4	47.0	47.4	47.7
R_{work} (%)	39.9	33.4	27.2	24.1	22.3	21.9
R_{free} (%)	46.6	38.2	32.8	28.6	27.2	26.7

[1] Numbers in parentheses refer to the highest resolution shell

Table S6 Data processing statistics for the CTXM beta-lactamase dataset, processed without ambigator. Data were collected at the T-REXX endstation on EMBL beamline P14 at PETRA III. Data were collected at 295 K. Samples were delivered to the X-ray beam using HARE-Chips (Mehrabi *et al.*, 2020).

**Data collection**						
# diffraction patterns	101	297	1008	3000	10009	27421
Space group	P3221					
Unit cell dimensions (Å, °)	41.9, 41.9, 232.8; 90.0, 90.0, 120.0					
Resolution range (Å)	116.3 – 1.70 (1.76 – 1.70)	116.3 – 1.70 (1.76 – 1.70)	116.3 – 1.70 (1.76 – 1.70)	116.3 – 1.70 (1.76 – 1.70)	116.3 – 1.70 (1.76 – 1.70)	116.3 – 1.70 (1.76 – 1.70)
Wilson B factor ($Å^2$)	18.6	25.8	24.5	24.7	24.4	24.2
# measured reflections	20412	84145	288693	893643	3106336	9720847
# unique reflections	7778	20809	27178	27451	27454	27455
Completeness (%)	28.3 (14.5)	75.8 (57.8)	99.0 (97.5)	100.0 (100.0)	100.0 (100.0)	100.0 (100.0)
Multiplicity	2.6 (2.3)	4.0 (2.9)	10.6 (6.5)	32.6 (19.7)	113.1 (68.6)	354.1 (214.3)
Signal-noise-ratio	2.4 (0.2)	1.6 (0.5)	1.5 (0.7)	2.4 (0.6)	4.2 (1.1)	7.3 (2.2)
Rsplit (%)	98.9 (89.6)	84.4 (314.3)	63.5 (307.1)	39.8 (182.6)	22.1 (94.0)	12.8 (46.6)
CC* (%)	49.8 (NaN)	68.1 (38.1)	86.2 (33.6)	93.6 (46.1)	97.8 (70.4)	99.2 (91.0)
CC1/2 (%)	14.2 (98.3)	30.2 (7.8)	59.2 (6.0)	77.9 (11.9)	91.8 (32.9)	97.0 (70.7)
**Refinement**						
Resolution range (Å)	77.6 – 1.70	77.6 – 1.70	77.6 – 1.70	77.6 – 1.70	77.6 – 1.70	77.6 – 1.70

Mean B (Å^2)	16.7	21.2	21.1	22.5	22.5	22.4
R_{work} (%)	40.9	34.7	26.0	19.9	15.4	13.7
R_{free} (%)	44.8	36.4	30.1	22.9	18.9	17.3
# Twin domains[2]	2	2	2	2	2	2

[1] Numbers in parentheses refer to the highest resolution shell

[2] As discussed in §3 of the main text, twin domains are erroneously detected by REFMAC throughout when *ambigator* is not used to resolve the indexing ambiguity.
